# Gamification in Initial Teacher Training to Promote Inclusive Practices: A Qualitative Study

**DOI:** 10.3390/ijerph19138000

**Published:** 2022-06-29

**Authors:** Ana Manzano-León, José Manuel Aguilar-Parra, Javier Rodríguez-Moreno, Ana María Ortiz-Colón

**Affiliations:** 1 Health Research Centre, Department of Psychology, University of Almería, 04120 Almería, Spain; aml570@ual.es; 2 Department of Education, University of Jaén, 23071 Jaén, Spain; jrmoreno@ujaen.es (J.R.-M.); aortiz@ujaen.es (A.M.O.-C.)

**Keywords:** educational innovation, gamification, higher education, qualitative study

## Abstract

Gamification consists of the use of ludic elements in non-ludic contexts. It is becoming an educational trend, due to its ability to work on curriculum skills in a fun and motivating way. This article exposes a program of gamified university practices, “Super-Profes”, for the subject of Developmental Disorders. To gain an understanding of student impressions about this methodology, a qualitative study was carried out, based on a survey with open questions, and, subsequently, analyzed with the Atlas.ti 8.4 program. In total, 63 s-year students taking the Early Childhood Education degree participated. Two main categories emerged from the study: gamification as a fun and motivating educational experience, and knowledge and skills acquired after studying a gamified subject. The research concluded with an assessment of educational gamification as a motivating and effective methodology for the acquisition of content and skills necessary for future teaching.

## 1. Introduction

University education should encourage its students to acquire new skills and tools that favor the future work performance of students [1]. In recent years, different methodologies have been put into practice that require university students to have a more active role in their teaching–learning process. Among these, the concepts of play and gamification as motivating elements in the classroom are being evaluated [2,3,4].

Gamification is defined as the use of game elements in non-playful contexts, such as the educational context [5]. Gamification pursues the use of game design elements to create engaging and motivating experiences [6]. The systematic review by Manzano-León et al. [7] indicates that gamification encourages greater motivation, academic performance and commitment, or engagement, in students. Among the most used elements in educational gamification are PBL (Points, Badges and Leaderboards), avatars and narratives [8]. 

Gamification can support pedagogical intervention as a playful, creative, and challenging learning strategy for students. It reinforces extrinsic motivation through external rewards, which, combined with a commitment to task and immersion, seek to achieve intrinsic motivation [9]. Diverse classroom experiences [2,10,11,12] show that gamification in the classroom was an effective tool to develop more experiential, participatory and creative activities, achieving greater flow and motivation from the students. In contrast, the possible risks of educational gamification must also be considered. Recent literature mainly mentions pointsification and excess extrinsic motivation [13,14]. When the gamification system, or PBL (Points-Badges-Leaderboard), consists primarily, or exclusively, of the acquisition of points for performing tasks, students may be interested in the short term. Still, it is very likely that they will only end up performing the tasks to get the points, subtracting intrinsic motivation and autonomy [15].

In initial teacher training, innovative teaching strategies serve a dual function. On the one hand, they promote curricular learning and university students’ motivation. For example, the qualitative research of Pérez-López et al. [16] shows how students studying for a Physical Activity and Sports Sciences degree perceived the use of gamification, namely, “The prophecy of the chosen”, which they described as significant and experiential learning, where they could have a relaxed classroom climate and feel greater responsibility in their evaluation process. These results coincide with other mixed qualitative–quantitative research, where it was reflected that, after the gamified practices of a music education subject in a Primary Education degree, the students experienced a high degree of involvement and collaboration, and had a greater predilection for game strategies linked to classifications, getting feedback and challenges [17].

On the other hand, the second function of teaching innovative learning strategies is to encourage future teachers to become familiar with teaching strategies that they can apply in their classrooms, since these strategies, and, specifically, gamification, can be integrated into the pedagogical design. It is necessary that teachers feel motivated to integrate it into their teaching practices. Today, a certain disinterest or resistance is sustained by beliefs about the usefulness of the use of games in education [18,19]. Gamification is not yet being implemented regularly in classrooms, although the teachers’ attitudes towards methodology are positive [20]. Valencia-Quecano and Orellana-Viñambres [21] highlight five barriers to using gamification: technology (cost, infrastructure, and technical errors); pedagogy (quality of content, assessment, and instruction); teachers (teaching attitude and lack of experience); students (lack of culture in collaborative work, experience, motivation, technology competence and learning difficulties) and design of the playful strategy.

The central purpose of this study was to investigate the opinions and reflections of Early Childhood Education degree students on the use of educational gamification, based on their participation in the gamified practices of a subject of their degree.

The following specific objectives were defined:

- Determine the benefits that the participants consider gamification brings to the teaching–learning process.
- Know the difficulties and disadvantages that students may perceive when undertaking a gamified subject.
- Explore whether educational gamification can promote awareness of functional diversity.

## 2. Materials and Methods

### 2.1. Participants

The initial sample consisted of 76 students of the degree in Early Childhood Education from a university in southern Spain where an educational innovation program was implemented through gamified practices. In the last internship session, each student volunteered to respond to an online survey of open-ended questions. This survey was completed by 63 students (82.9% of participants), 59 women and 4 men, aged between 19 and 31 years (M = 21.7; DT = 4.7).

The method for choosing participants was intentional non-probabilistic sampling. The inclusion criteria to participate in the study were the following: studying the subject Developmental Disorders (a compulsory subject in the second year of the Early Childhood Education degree), taking this subject during the academic year 2019/2020 and having an attendance higher than 50% of the practical sessions.

### 2.2. Instrument

An exploratory and descriptive research design was carried out, based on qualitative research in Campo [22], through an open-ended questionnaire. Open-ended questions seek to record arguments with a high degree of detail [22].

The proposed instrument to obtain students’ impressions was a structured and open interview by means of a Google form in an online format. For the selection of questions, an initial survey was designed that was validated by expert judgment, obtaining a positive assessment. The survey was then applied to five university students to ensure understanding of the questions. After changing two questions to ensure better understanding, the final version was distributed, and was composed of the following questions (See Table 1).

The thoughts and reflections of the students participating in the gamified practices were revealed from their answers to the proposed questions.

The intention of encouraging elaboration with these questions was related to the purpose of gaining an understanding of the participating students’ experiences of gamification practices and their experiences concerning the teaching–learning process.

The online modality favoured anonymity as a form of expression, which allowed students to express themselves freely and to explore their responses in depth [23].

### 2.3. Data Analysis

All surveys were grouped into a document for transcription. The analysis process was emergent, respecting the opinions and beliefs constructed by the participating students. 

After unifying the answers, the first open coding was performed. It consisted of carefully examining the data to identify the meaning of the students’ answers, and to conceptualise the first quotes and codes of importance. Next, axial coding was carried out, where the categories and subcategories of the stories were related. Finally, in selective coding, the categories were organised into tables. These phases were carried out through the discussions of two researchers, and, in the case of disagreement, a third researcher was contacted.

The document was analysed with ATLAS.ti software (Version 8.4., ATLAS.ti Scientific Software Development GmbH, Berlin, Germany).

### 2.4. Procedure

A gamified methodology, SuperProfes, was designed and evaluated for the compulsory subject of “Developmental Disorder” in the second semester of the second year of the degree in Early Childhood Education in the academic year 2019/2020. The specific competencies of this subject are: (1) Identify learning difficulties, cognitive dysfunctions, and relationships with attention; (2) Know how to inform other specialist professionals to address the collaboration of the centre and the teacher in the attention to the special educational needs that arise; (3) Acquire resources to promote the educational integration of students with difficulties. In addition, as part of the hidden curriculum, it is vital to teach and raise awareness about functional diversity among future teachers, since teachers are crucial to promoting the participation and learning of all students, thus, promoting inclusion and equity [24].

A gamified methodology was implemented in the practical classes to promote students’ learning and awareness through motivating practices.

“Super-Profes” consisted of a gamification and game-based learning program of 8 weekly sessions, each session having a duration of two and a half hours. The gamification was structured within the MDE model (Mechanical-Dynamic-Aesthetic) [25], where the following elements were proposed:

- Aesthetics: Superhero narrative
- Dynamics: Competition and challenges
- Mechanics: Medals, points, ranking and virtual reward

This project was part of a cooperative Project-Based Learning (PBL) methodology, which implemented a set of tasks that pose a challenge for students [26,27,28].

It contained a narrative where the students were part of a super academy and had to transform into superheroes and save the world from the evil Clone Usixe (an acronym for exclusion). To do this, they had to create teams of 4 to 6 students and work cooperatively. To defeat the enemy, it was proposed that they had to solve three challenges, which were evaluated and qualified through the use of rubrics: First, a criticism of a film whose protagonist character had a disability, or where the theme of the film was related to a disability; Second, an awareness practice, where students had to make a 2 to 5 min video on functional diversity; Third, teams had to create a virtual learning pill, with each team of students being given a type of disability and tasked with creating theoretical content, as well as researching innovative resources and methods, to work on the difficulties related to the assigned disability.

**Figure 1:**
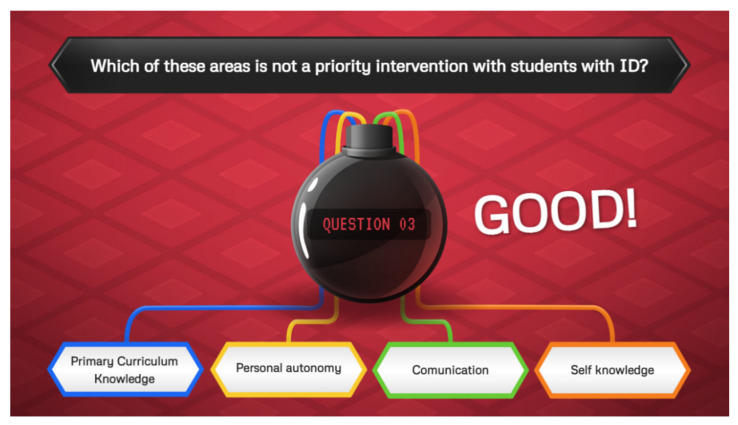
Quiz on the syllabus of the subject in the escape room.

**Figure 2:**
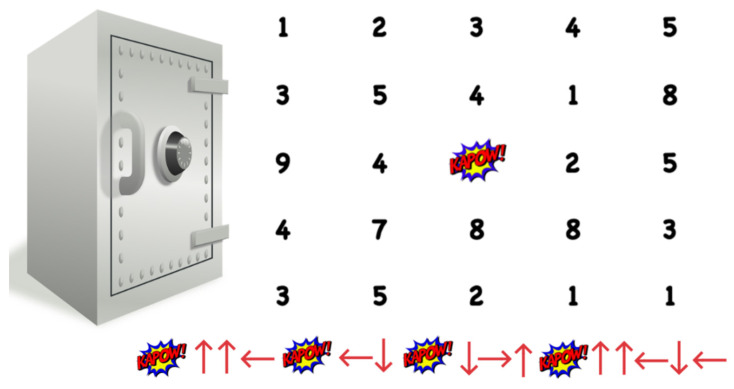
Playful challenge (Puzzle) in the escape room. The puzzle is solved by following the arrows from the “KAPOW!” icon, giving 2723 as a solution.

The learning pill also had to contain some challenges related to disability. Some examples were learning a song in sign language, creating a message in braille, and writing your name in a mirror with your non-dominant hand. When a team of students performed the challenge, another team could win a virtual medal. These virtual medals could add up to 0.5 in the practice section of the subject, and if the complete class got 15 medals (an average of 2.5 medals per team), a surprise activity was unlocked. This activity consisted of an online escape room related to the content of the subject (See Figure 1 and Figure 2). Escape rooms were immersive games played by small groups of three to eight people, in which participants were required to solve puzzles to escape from the room or, in the case of a breakout, open a final chest [29]. Specifically, the escape room aimed to be the narrative and lucid closure of the practices of the subject, a place to review the subject and defeat the enemy of gamification. The escape room had five challenges to be completed:

1. Didactic challenge: Quiz about the content of the subject
2. Playful challenge: Puzzle and guess the secret word
3. Didactic challenge: Quiz on the content of the subject
4. Playful Challenge: César Code and guess the secret word
5. Final challenge: Escape from a class by means of, first, opening a lock of addresses thanks to a message in Braille and the elaboration of two school diagnoses and, second, a puzzle to open a safe combination.

The escape is located online in Spanish at the following address: https://diversatics.wixsite.com/mision (accessed on 26 June 2022). 

This methodology was implemented in the second semester of the academic year 2019–2020, so all the proposed activities were carried out online. To seek collaborative and student-centred models, team activities were proposed synchronously online, although asynchronous collaboration was accepted in exceptional cases due to situations caused by COVID-19, such as having to share a personal computer with other family members and having internet connection problems [30].

The teaching role during the subject consisted of the role of pedagogical accompaniment [31], wherein, during each session, the doubts of each team of students were oriented.

The gamification was designed jointly by the teacher coordinator of the subject and one of the leading researchers. Before the implementation, the teacher carried out a three-hour training on playful learning strategies. The necessary resources were provided for gamification.

Before data collection, students were informed of the nature of the study and their anonymity was assured. The whole process was carried out following the Declaration of Helsinki. Ethics approval was obtained from the Research Ethics Committee of the University of Almería (Ref. UALBIO 2021/01). All participants provided their oral and written informed consent for participating in the study and the subsequent publication of anonymous responses.

## 3. Results

After analysing the surveys completed by the students who carried out their gamified practices, the following categories were identified (See Table 2 and Table 3). To respect the anonymity of the students, each person is determined by an E and a number chosen for each student in the linear order of receipt of the surveys.

### 3.1. Gamification as a Playful and Motivational Educational Experience

First, the results show how students perceived the gamified environment through the MDE (Mechanical-Dynamic-Aesthetic) model as a fun and motivating way of working. 

The students highlighted the challenges in a general way (the practice of awareness, the report of films related to functional diversity, the learning pill and the overcoming of challenges launched by other teams) and, specifically, the escape room. In the case of motivation towards challenges, Pietro Andreu [32] states in his systematic review that gamification promotes extrinsic and intrinsic motivation thanks to this type of challenge, which arouses the curiosity of university students, allows some control to the student, and contains elements of fantasy. The challenges of our gamification were integrated into the superhero narrative. However, it was observed that the narrative was not mentioned much as a remarkable element. This fact made our results differ from other research where students prioritised the narrative as an element of engagement [33,34]. The students in our study were mainly motivated by the practical nature of the dynamics and mechanics, as well as by the playful nature of the challenges.

The escape room received good reviews from students, reaffirming that it could be an interactive and motivating activity that served as a reinforcement of the university curricular content. Dietrich [35] reinforces this idea, as their study shows how an escape room in the classroom encourages students to work on concepts playfully and cooperatively and allows them to develop adaptive and receptive skills, compete with and against their peers, show their individual skills, interact with each other, and experience moments of discovery and victory.

Secondly, regarding gamification as an educational methodology, the participating students felt that gamification had great potential in education. Most students mentioned that “Super Profes” was an active, dynamic, and motivating internship program through which to understand content. In addition, they demanded that more traditional subjects be reduced to give way to other more significant methodologies in their learning process. These results coincide with previous qualitative research [16], where students, after completing a gamified subject, showed high satisfaction in the acquisition of significant learning and a climate of positive coexistence that could favour prosocial skills.

Finally, it was highlighted that the students did not mention possible improvements concerning the proposed playful elements. The aspect of progress that they suggested was the lack of face-to-face classes which could be improved. Due to the academic year where this research was carried out, it was impossible to modify, and individual activities were included along with group activities. In future implementations of the program, these results will be considered, and at least one of the challenges will be proposed individually.

### 3.2. Knowledge and Skills Acquired after a Gamified Subject

The general results of this category showed that the students considered the gamified practices of the subject had been very positive with respect to their learning, competences, and awareness.

The acquisition of meaningful learning can be directly related to motivation and commitment to learning. Educational gamification, specifically at the university stage, requires its design to be focused on tasks that students consider useful in their training, and that enrich them with playful elements, since the effectiveness of the gamified system lies in the motivation it manages to maintain in the user [36]. To achieve a long-term commitment it is necessary that a system favors mechanisms related to intrinsic motivation, that is, challenges that students perform not only for the final grade, or for some reward of the game system (extrinsic motivation), but for the self-interest of learning and/or enjoying the task [37]. Here it was evident that the gamification methodology was not only playful, because it had purpose beyond fun. Gamification promoted changes in the participants (behavioural, participation, commitment, engagement, and/or learning). Gamification has, as its primary objective, a goal to influence the behaviour of people, regardless of other secondary objectives, such as enjoyment during the performance of the gaming activity [38].

Finally, the students mentioned that performing gamified challenges related to the theme of functional diversity and developmental disorders fostered a greater understanding of abilities and limitations, thus achieving greater empathy towards developmental disorders. To complete an inclusive education, future teachers must be trained to be sensitive to the needs of all students so that they can promote inclusive educational processes in their future teaching work [39].

## 4. Discussion

The use of playful educational strategies, specifically in the university environment, is an educational trend. In general terms, its primary benefits are observed to be greater motivation of students, positive willingness to learn and immediate feedback [40]. Its most significant risks are excessive use of extrinsic rewards that do not favour the intrinsic motivation of students and an adverse classroom climate, due to competitiveness [41]. 

This study argues the possibilities of gamification to support other active learning methodologies, such as challenge-based and cooperative learning. Our results match previous studies [42,43,44], demonstrating that gamification and project-based learning ensure that students have excellent academic performance and feel motivated with the subject. In addition, cooperating with other students favours collaborative and prosocial skills, key competencies necessary for future teaching work [45].

Proposing cooperative activities has been a challenge in recent academic years due to COVID-19 protocols and hygienic measures for the prevention of the spread of the virus strongly discouraging any contact. As a consequence, there has been a generalised distance between students, teachers, and administration, which generated situations of stress and overflow [46]. After participating in the gamified practices, it could be concluded that, despite the general demotivation because the classes were online and they could not work in person, the students considered the gamified practices playful, pleasant, and a formative learning experience. The application of gamification has meant more significant time and teacher involvement since the design requires an initial effort greater than that necessary in traditional education [47]. Still, its benefits have been demonstrated in the learning of university curricular content, showing itself to be an effective tool to facilitate a mixed modality, which can be adapted to the needs of the students. However, the importance of providing university teachers with training and resources to apply this methodology and to adapt educational environments to future challenges related to online learning environments requires discussion.

The results of this research represented the perceptions of 63 university students of an Early Childhood Education degree, where the possibilities and limitations of gamification and learning, based on cooperative challenges, were analysed. However, this study had several limitations. First, the limited number of the sample is noted, so the conclusions cannot be generalised. Second, since there were no interviews, it was impossible to delve into students’ responses. Finally, the lack of a combination of quantitative and qualitative methods that could favour the validity of the research is relevant. For these reasons, future studies are needed to extend the experience and research on playful methodologies to other degrees and universities and investigate their effects on other psycho-pedagogical variables.

## 5. Conclusions

In conclusion, university teachers should seek pedagogical strategies that facilitate their students’ acquisition of curricular content and professional skills. In this research, the use of gamification in the practices of the Early Childhood Education degree was evaluated. This research concludes that gamification was valued as a positive tool for its ability to motivate and engage students with the proposed tasks and, in turn, fostered learning and the positive predisposition of students towards the subject.

## Figures and Tables

**Table 1:** Survey questions.

Theme	Questions
I: Sociodemographic	Degree, course, age, and sex
II: Opinion about cooperative gamification	(1) Did you like how the subject was proposed? Why? (2) How have you felt about working in a group? Would you have preferred to work individually? (3) How do you rate the teaching team? (4) What did you like most about the subject?
III: Learning and awareness	(5) What do you think you have learned in this subject? (6) Do you think you will know how to act if you have students with SEN in your class?
IV: Limitations and proposals for improving gamification	(7) Is there anything you did not like about Super-profes? (8) Have you had any difficulty doing the internship?
V: Others	(9) Are there any other comments you want to mention about Super-Profes or the subject?

**Table 2:** Results of main category 1.

Main Category	Subcategory	Frequency	Citation Example
Gamification as a playful and motivating educational experience	MDE	55 from 65, 87.3%	I loved how this subject has been proposed. Because I’ve had a great time doing the challenges. (E 3) The activities, such as the pill, creating the video or the challenges of the companions were a way to see your companions in these moments and have a fun time. (E 17) The escape room has been very cool, not only because it was the first time but because it has kept us totally in tension until the last moment. In the end it has been the finishing touch of the subject, a clear reflection of everything lived throughout the semester. I can only say, thank you. (E 21)
	Motivation	60 from 63, 95.2%	[I liked it] a lot, because the teacher has raised it in a very bearable playful way and without any pressure. It has made us like it when doing the internship, etc. (E 40) The activities were entertaining and motivated me to learn much more. (E 68)
	Fun	59 from 63, 93.6%	I liked it very much since the subject has been raised in a very dynamic and playful way in which the classes have not bored us. (E 28)
	Active methodology	60 from 63, 95.2%	There have been many activities that have allowed us to develop in an entertaining and practical way the contents of the subject. (E 3) I found the approach super dynamic, nothing heavy and even fun. It gives a place to theory because it must have its space, but also to practice and dynamism. I personally love working with practical things rather than just having theory, a good mix is key. (E 19)
	Different from traditional education	19 from 63, 30.2%	I loved it, because it is not the traditional approach that the rest of the teachers follow to put some slides and explain them, that in the long run becomes boring and monotonous. With this approach, through the activities we have been doing, we have learned the contents of this subject in a more enjoyable and fun way. (E 25) Super profes has been totally different from the other [subjects] and I think that all teachers should rethink making their subjects as meaningful as this one. (E 30)
	Limitations of online gamification	5 from 63, 7.9%	I have missed the face-to-face classes, being able to interact better with the teacher and my classmates. (E 51) On some occasions I would have preferred individually, but it is true that in a group we have been able to help each other. (E 34)

**Table 3:** Results of main category 2.

Main Category	Subcategory	Frequency	Citation Example
Knowledge and skills acquired after a gamified subject	Specific knowledge related to the subject (Developmental Disorders)	61 from 63, 96.8%	[I have learned] all kinds of needs, supports, and actions before the different types of disorders and difficulties. (A 1) I have learned many things about the disorders since I knew them but not thoroughly. (A 24) I have learned many things about all the developmental disorders that exist, I find it very interesting to know so many things about them, what they are, when they occur, how to act… (E 57)
	Learning for teacher skills	26 from 63, 41.3%	Thanks to the subject I have been able to acquire and know guidelines and strategies for the future as a teacher on how to act if there is a child with a disorder in the classroom. (E 16) My knowledge related to developmental disorders was very non-existent and through it [the subject] in the future I will understand and understand much better any student with their own characteristics. (E 30) I have learned new things that I know in the future will be useful to me as a teacher in the nursery classroom. (E 31)
	Cooperative work skills	48% from 63, 76.2%	I have loved working in a group since we have learned from each other, and I think it has helped us to also know how to listen to different opinions and agree. (E 11) I have learned mainly to cooperate with my classmates, I have learned to have fun while I was learning and that has made me learn with more enthusiasm. (E 28)

## Data Availability

The data presented in this study are available on request from the corresponding author.
